# The Thermoregulatory Function of Thatched Nests in the South American Grass-Cutting Ant, *Acromyrmex heyeri*

**DOI:** 10.1673/031.010.13701

**Published:** 2010-08-23

**Authors:** Martin Bollazzi, Flavio Roces

**Affiliations:** Department of Behavioural Physiology and Sociobiology (Zoology II), Biocenter, University of Würzburg

**Keywords:** building behaviour, thermal biology, nest material, heat transfer, leaf-cutting ants

## Abstract

The construction of mound-shaped nests by ants is considered as a behavioral adaptation to low environmental temperatures, i.e., colonies achieve higher and more stables temperatures than those of the environment. Besides the well-known nests of boreal *Formica* wood-ants, several species of South American leaf-cutting ants of the genus *Acromyrmex* construct thatched nests. *Acromyrmex* workers import plant fragments as building material, and arrange them so as to form a thatch covering a central chamber, where the fungus garden is located. Thus, the degree of thermoregulation attained by the fungus garden inside the thatched nest largely depends on how the thatch affects the thermal relations between the fungus and the environment. This work was aimed at studying the thermoregulatory function of the thatched nests built by the grass-cutting ant *Acromyrmex heyeri* Forel (Hymenoptera: Formicidae: Myrmicinae). Nest and environmental temperatures were measured as a function of solar radiation on the long-term. The thermal diffusivity of the nest thatch was measured and compared to that of the surrounding soil, in order to assess the influence of the building material on the nest's thermoregulatory ability. The results showed that the average core temperature of thatched nests was higher than that of the environment, but remained below values harmful for the fungus. This thermoregulation was brought about by the low thermal diffusivity of the nest thatch built by workers with plant fragments, instead of the readily-available soil particles that have a higher thermal diffusivity. The thatch prevented diurnal nest overheating by the incoming solar radiation, and avoided losses of the accumulated daily heat into the cold air during the night. The adaptive value of thatching behavior in *Acromyrmex* leaf-cutting ants occurring in the southernmost distribution range is discussed.

## Introduction

In insects, long-term adaptation to changing or adverse climatic conditions may adopt the form of physiological responses such as diapause, aestivation, or dormancy, but short-term adaptations mostly adopt the form of behavioural responses (Chown and Nicolson 2004). Behavioural responses are often the primary, and sometimes the crucial, means by which an animal copes with environmental changes (Bennet 1987). For social insects, the outcome of their collective building activities has been considered the main way by which colonies gain some degree of control over the environment (Hansell 2005). In ants inhabiting temperate zones of the northern hemisphere, the construction of moundshaped nests is considered as a behavioral adaptation to low environmental temperatures, i.e., the mound helps the colony to achieve higher and more stables temperatures than those of the environment (see reviews in Gösswald 1989; Heinrich 1993).

Mound-building ants also occur in the southern hemisphere and include, besides *Camponotus* and *Solenopsis* ants, at least seven species of the leaf-cutting ant genus *Acromyrmex* (Bonetto 1959; Weber 1972; Gonçalves 1961; Fowler and Claver 1991; Seeley and Heinrich 1981). Some mound-building *Acromyrmex* often show intraspecific nesting plasticity, i.e., colonies may inhabit either a mound or a subterranean nest (Fowler 1985; Diehl-Fleig and Droste 1992; Guerra de Gusmão 1998; Gonçalves 1961). Moundbuilding behavior in *Acromyrmex* has also been discussed as an adaptation to low environmental temperatures. The symbiotic fungus cultivated by leaf-cutting ants, which represents the sole food source for the developing larvae, requires high humidity and temperatures between 25 and 30° C for proper growth (Powell and Stradling 1986). It has been hypothesized that the ants maintain conditions in the nest that promote growth of the fungus. Three lines of evidence emphasize the thermoregulatory benefits of moundbuilding in leaf-cutting ants. First, colonies of *Acromyrmex* species inhabiting mound-shaped nests have a more southerly distribution than those living in subterranean nests (Farji-Brener 2000). Second, species showing nesting plasticity build superficial moundshaped nests in cold soils, yet excavate subterranean nests in warmer ones (Bollazzi et al. 2008). Finally, several observations show that average internal nest temperature inside mound *Acromyrmex* nests is higher than that of the environment (Farji-Brener 2000; Quiran and Pilati 1998; Zolessi and Abenante 1973; Zolessi and González 1974). Thus, *Acromyrmex* mounds in the southern hemisphere appear to have the same function as the *Formica* mound-shaped nests in the northern hemisphere (Farji-Brener 2000).

For mound-building ants in general, both solar radiation reaching the exposed nest and metabolic heat produced either by colony members or the decaying nest material, have been shown to be major heat sources influencing nest temperature (Frouz 2000; Horstmann 1990; Coenen-Stass et al. 1980; Rosengren et al. 1987; Seeley and Heinrich 1981). Whatever the origin of heat, the longterm maintenance of a higher and more stable inside-nest temperature would largely depend on the way the mound material influences heat flow between the nest interior and the environment.

Mounds built by ants in the northern hemisphere are often permeated with galleries,that allow them to move the broodto areas with appropriate temperatures (Heinrich 1993). Dense mounds made of soil and plant debris are also known in *Camponotus spp.* and *Solenopsis spp.* inhabiting southern South America (Bonetto et al. 1961; Folgarait et al. 2002; Tschinkel 2006). Conversely, mounds of *Acromyrmex* leaf-cutting ants, built with plant fragments and debris, are arranged by workers so as to form a thatch with a single central chamber for the fungus i.e. where the fungus and brood are not located within the thatch structure, but enclosed by the thatch material. This restricts the occurrence of fungus relocation as a thermoregulatory response, so that the achievement of a proper temperature for colony growth largely depends on how the thatch affects the heat exchanges between the fungus garden and the environment.

This work was therefore aimed at studying the thermoregulatory function of the thatch in the mound nests of grass-cutting ants, *Acromyrmex heyeri* Forel (Hymenoptera: Formicidae: Myrmicinae). For that, the contribution of both the incoming solar radiation and colony's inhabitants on nest temperature was investigated in field nests. How the thermal properties of the thatch material influences the thermal relations between the fungus garden and the environment was also examined.

## Materials and Methods

This study was performed on colonies of the grass-cutting ant *Acromyrmex heyeri* occurring in temperate grasslands of southern South America (Fowler and Claver 1991). Nests were located in a farm in Joanico, south Uruguay (34° 33′ 26″ S; 56° 15′ 59″ W).

### The effect of incoming solar radiation and colony presence on fungus temperature of thatched nests

The effect of the incoming solar radiation on fungus garden temperature was studied both in the long- and the short-term. In the longterm, by assessing the relationship between fungus temperature and the daily accumulated direct solar radiation throughout the year. In the short-term, by quantifying the effects that an experimental nest shading precluding direct solar radiation have on fungus garden temperature.

To control for the differences in thatch thickness among colonies, which are expected to influence the dynamics of the temperature change inside the nest, long- term measurements were restricted to single thatched nests. These measurements were compared to simultaneous measurements performed in a similar-sized fungus garden of a neighbor colony inhabiting a subterranean nest, as well as to the environmental temperature. Measurements were performed from mid winter to early summer, when the incoming solar radiation shows a stepper yearly increase in the southern hemisphere (Rosenberg et al. 1983). The first measurement period started in June 27^th^ 2001 in a thatched and a subterranean nest. Both contained a single fungus garden, 30 cm in diameter. While the fungus garden of the thatched nest was located at 15 cm depth and was covered by a thatch of 8–10 cm thickness, the bottom of the subterranean nest was at 45 cm depth. This first measurement period lasted until January 24^th^ 2002, when the thatched colony moved. From July 25^th^ until November 12^th^ 2002, a new pair of a thatched and subterranean nests were investigated. They were similar in size, chamber depth and thatch thickness to those monitored in 2001. Fungus garden temperatures of both nests were simultaneously measured in the central portion of the fungus gardens. This was done by inserting a sensor in the middle of the fungus garden, which was connected via a 2 m long cable to a data logger placed outside the nest. Air temperature was measured at 1 m height, and soil temperature at 2–3 cm depth. Temperatures were recorded every 30 minutes using Tinytag (www.geminidataloggers.com) data loggers, and downloaded to a notebook every week.

The daily accumulated income of direct solar radiation (kJ*day^-1^*cm^-2^) was estimated using the Angström equation modified by Page (Goswami et al. 2000): R_s_ = R_0_ (a+b n/N), where R_s_ and R_0_ are the horizontal terrestrial and extraterrestrial radiation levels averaged for 24 h, n is the daily measured hours of sunshine, N is the daily astronomically possible hours of sunshine, a and b are constants experimentally obtained for the study area, which were take from Corsi (1980). Daily values of n, N, and R_0_ for south Uruguay were obtained from the GRAS program of the Ministry of Agriculture of Uruguay (INIA-MGAP 2007). Mean soil temperatures at 50 cm depth were calculated for the study area for winter, spring and summer. The Newhall Simulation Model used by Van Wambeke (1981) was used to determine South American soil temperature regimes. Daily mean air temperatures for the years 1972 to 2001, obtained from Las Brujas Meteorological Station, situated 15 kilometers west of the study area, were used for calculations (INIA-MGAP 2007).

In the short-term, the effect of incoming solar radiation on fungus garden temperature was evaluated by comparing fungus chamber temperatures in experimentally-shaded nests, with the temperatures measured in the same nests before and after shading. Four thatched nests were covered by a an 80 × 80 cm wooden roof for 20 days, placed 15 cm above the mound top. A 30 cm-thick layer of dry plants was placed on the wooden plate to avoid direct heating of the roof by sunshine. Fungus chamber temperature was logged at 30-min intervals, as described above, for one week before the roof was installed, the 20 days of shading, and one week after removal of the roof.

The contribution of the colony (fungus garden and ants) to the nest temperature was evaluated by measuring nest temperatures before and after the inhabiting colony was poisoned. For that, four thatched nests were first covered by a roof for 20 days as described above, to control for the effects of solar radiation. Simultaneously with the placement of the roof, the colonies were poisoned using a commercial citrus-pulpbased bait containing 0.03% Sulfluramid® (www.lanafil.com). Fungus chamber temperature was logged for one week before the roof was placed, i.e., before the chemical treatment, for 20 days after the poisoning, in which nests were shaded, and for a week after removal of the roof. This experiment was carried out as a part of the annual pest management performed by local farmers during the summer 2003. Two days after being poisoned, colonies stopped cutting grasses and died after one week, yet the thatch structure remained undamaged.

### Thermal properties of thatch and soil

To explore how the building material influences the thatch's thermal properties, the thermal diffusivity of a natural nest thatch built with grass fragments was determined, and compared to that of the soil surrounding the nest. The thermal diffusivity of a given material is the ratio between the heat it conduces, which depends on its thermal conductivity (k), and the amount of heat it stores per unit volume, which depends on its heat capacity (C). The larger the thermal diffusivity, the faster the propagation of heat into the medium (Cengel 2003). Therefore, if the thatch material facilitates heat exchange between the fungus and the environment, a high thermal diffusivity of thatch material should be expected. On the contrary, a low thermal diffusivity should be expected if the thatch material limits heat exchanges.

In order to measure the thermal diffusivity of the thatch, ca. 500 cm^3^ of thatch material were collected every 2 days from the upper portion of a single thatched nest. It was done for two weeks during March 2005, totaling 8 thatch samples. Simultaneously, samples of the soil surrounding the nests were collected. Each of these soil samples consisted of a mixture of ca. 30 cm^3^ taken at 2, 10 20 and 40 cm depth. Immediately after collection, the water mass content of all thatch and soil samples was measured using a gravimetric method (Smith and Mullins 2001). Thermal diffusivity was measured at the Department of Behavioural Physiology and Sociobiology at the University of Würzburg, Germany, using a Dual Probe Heat Capacity Sensor (see below). Since water content influences thermal diffusivity (Campbell 1977), the relationship between thermal diffusivity and water content of the thatch material and surrounding soil was measured. Therefore, soil and thatch samples were milled and dried for 1 week at 50°C. Thermal diffusivity was measured in the dried samples, assigned as having 0% water mass content. Afterwards, water was added to the samples until they successively reached 10 20 and 30% water mass content. Thermal diffusivity was successively measured in the probes with different water contents.

A Dual-Probe Heat Capacity Sensor (DPHC) was constructed for the measurements (Campbell et al. 1991). Thermal diffusivity was calculated by dividing thermal conductivity by heat capacity. The DPHC consisted of a small plastic body with a heater and a thermocouple placed parallel to each other. The heather was a ceramic pt100, (Pt100 “Messwiderstand”, Electhortherm®, (www.electrotherm.de) of 10 mm length, 2 mm width and 0.5 mm thickness; the sensor was a wire sensor (Typ K NiCrNi, B+B Thermo-Technik, (www.bubthermo.de) connected to a data logger (Voltcraft® K204, Conrad Electronics, www.conrad.de). The heater and the sensor were inserted into the material to be tested, which tightly filled the space between them.

A brief current pulse (5s) was applied to the heater, and the temperature of the thermocouple was monitored with the data logger every second. The response of the thermocouple to the heat pulse was used to simultaneously determine the heat capacity, C, and thermal conductivity, k, of the probe (Ham 2001; Ham and Benson 2004; Campbell et al. 1991). The heat capacity (J*kg^-1^*°C^-1^) was calculated using:

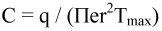

where q is the applied power per length of heater probe (J/m), r is the distance between the heater and the sensor (m), and T_max_ is the temperature increase at the thermistor probe (°C). The heat loss to the opposite side of the thermocouple, due to the use of a plate-shaped heather instead of a cylindrical-shaped one, was corrected by dividing the numerator q by 2. The thermal conductivity of the soil (W*m^-1^*°C^-1^) was calculated using:

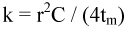

where C is the calculated heat capacity and t_m_ (s) is the elapsed time required to reach T_max_. The duration of the pulse (5s) was controlled by a computer-switched relay board placed between the current source and the heater (Relay board eight-fold serial, Conrad®). After a pulse, a new one was applied only after the sample temperature decreased again to room temperature, 23° C. Five pulses were applied to each of the 8 soil and thatch samples, for each water mass content, totalizing 40 measurements pro water mass content.

The DPHC calibration was performed using water-free glycerin at 23° C as a standard reference material. Thus, the obtained values of C and k for the water-free glycerin (C = 2.216 J*kg^-1^*°C^-1^, SE = 0.054; k = 0.31 W*m^-1^*°C^-1^, SE = 0.009) were similar to those obtained by Fontana (1999) using a DPHC (at 23° C, C = 2.215 J*kg^-1^*°C^-1^; k = 0.29 W*m^-1^*°C^-1^), and those given by Rahman (1995) (at 27° C, C = 2.42 J*kg^-1^ *°C^-1^· k = 0. 286 w*m^-1^*°C^-1^) and Cengel (2003) (at 20° C, C = 2.31 J*kg^-1^*°C^-1^).

**Figure 1.**
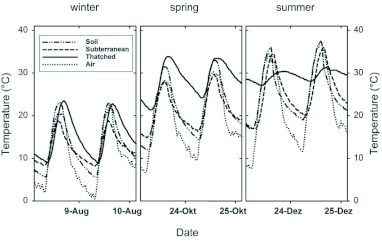
Examples of temperature variation in fungus gardens of a thatched and a subterranean nest of *Acromyrmex heyeri,* as well as in the soil, over 48 h in winter, spring and summer 2001. High quality figures are available online.

## Results

### The effect of incoming solar radiation and colony presence on fungus temperature of thatched nests

Figure 1 presents examples of the daily variation of temperature over 48 h, recorded for the thatched and subterranean field nests in winter, spring and summer. While the temperatures in the subterranean fungus roughly corresponded to that of the soil, the thatched fungus showed a different pattern. The amplitude of the daily temperature changes was observed to decrease from spring to summer, with daily maximal temperatures in summer below those of the soil, air, and the subterranean fungus.

**Figure 2.**
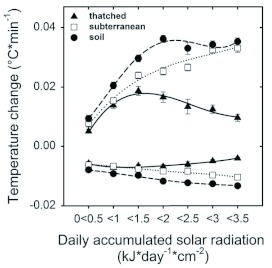
The rate at which temperature increased between daily minima and maxima during the day (upper portion), and decreased between daily maxima and next-day minima at night (lower portion), in the thatched fungus (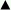), the subterranean fungus (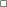), and the soil (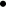), as a function of daily accumulated solar radiation (categories) (mean ± SE, N = 292 days). High quality figures are available online.

The daily variation of temperature depicted in Figure 1 for both nest types and the soil depended on the magnitude of heat gains and losses throughout the day. During the day, the incoming solar radiation drove the temperature of the soil and both nest types to its daily maximum. At night, the rate at which the gained heat was lost to the cooler environment (the air) determined the minimal temperature reached at the beginning of the following day (next-day minimum). Being more exposed, a thatched nest was a priori expected to experience steeper heat gains than the subterranean nest, as well as a faster nightly loss of any gained heat. Contrary to this assumption, the thatched fungus garden gained heat at a rate similar to that of the subterranean nest and the soil for low values of incident solar radiation (< 1 kJ*day*cm^-2^), yet at a lower rate for mid and high values (Figure 2, upper portion). Furthermore, the thatched fungus lost its gained heat at night at a slower rate than the subterranean fungus and the soil (Figure 2, lower portion). In general, the rates of temperature decrease were low and slightly dependent on solar radiation.

**Figure 3.**
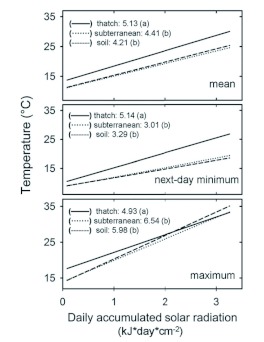
Regression lines of the daily mean, the daily next-day minimum, and the daily maximum of the thatched fungus (continuous line), the subterranean fungus (dotted) and the soil (dashed) as a function of daily accumulated solar radiation. All regressions are significant at p<0.01 (T-test) (N = 292 for each regression). The slopes are given in the insets of each graph. Regression slopes followed by the same letter are not significantly different. T-test, at p<0.05 for daily means, at p<0.01 for next-day minimum and maximum (Fowler et al. 1998). Regression coefficients of the thatched, subterranean and soil for the daily means: 0.44, 0.50, 0.51; for the next-day minima: 0.38, 0.26, 0.37; for the daily maxima: 0.43, 0.70, 0.64. High quality figures are available online.

The average, maximal and minimal temperatures reached by the thatched nest ultimately depend on the rates of heat gain and loss, as shown above (Figure 2). Figure 3 shows these values as a function of the daily accumulated income of direct solar radiation.

The slope of the daily mean as a function of solar radiation was slightly steeper for the thatched fungus than for the subterranean fungus and the soil. This indicates that the surplus of temperature observed in the thatched fungus increased with increasing solar radiation (Figure 3, upper portion). The slope of the next-day minima as a function of daily solar radiation was also significantly steeper for the thatched fungus than for the subterranean fungus and the soil (Figure 3, middle portion,). It follows that the extent to which the next-day minimum of the thatched fungus surpassed that of the subterranean fungus and the soil directly depended on the daily incoming solar radiation. However, the slope of the daily maxima for the thatched nest was significantly lower (Figure 3, lower portion legend). Although the thatched fungus and soil maxima were positively related to solar radiation, the daily maxima of the thatched fungus exceeded that of the soil, and therefore that of the subterranean fungus as well, only for a low accumulated solar radiation (< 1.5 kJ*day*cm^-2^), being equal or lower at greater values.

**Figure 4.**
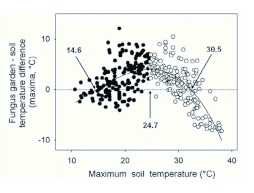
Temperature difference between the fungus garden and soil for the thatched nests (daily maxima), as a function of maximum soil temperature. The curved line is the best-fit polynomial regression line; f(x) = -5.93-0.17x+0.06x^2^-0.001 6x^3^; R^2^ = 0.46, p<0.01 (Fowler et al. 1998). Temperature values marked by the arrows show the soil T (° C) at which f(x) = 0, i.e., the soil temperature at which there was no temperature difference between soil and fungus. (14.6 and 30.5° C). The soil temperature at which the highest temperature difference between soil and fungus occurs was obtained by setting the first derivative to zero, f (x) = b+2cx+3dx^2^ = 0, and is indicated in the graph (24.7 °C). The black points (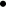) represent those values at which f(x) > 0, R^2^ = 0.23, b = 0.48; p<0.01. The white points (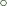) represent those values at which f(x) < 0, R^2^ = 0.56, b = 0.75; p<0.01. High quality figures are available online.

The relationship between the maximal temperatures reached daily by both the soil and the thatched fungus is shown in Figure 4. The extent to which the fungus daily maximum differed from that of the soil is expected to result from the different rates of heat losses and gains in fungus and soil, as depicted in the Figs. 2 and 3. The difference between the fungus and soil daily maxima was observed to continuously increase up to a maximal soil temperature of ca. 24.7° C. Beyond this value, the difference decreased and turned out to be opposite for maximum soil temperatures higher than 30.5° C, i.e, fungus and soil maxima were positively related only for soil maxima lower than this value of ca. 24.7° C, yet negatively correlated for higher values (Figure 4, statistics are in the figure legends). For maximal soil temperatures higher than 30.5° C, the difference became negative (Figure 4), i. e., the maximal temperature of the fungus garden became lower than that of the soil.

**Figure 5.**
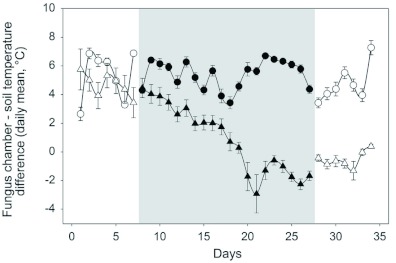
Temperature differences between fungus chamber and soil (daily mean ± SE, four nests each point) before the nests were shaded (before), during shading (shaded) and after shading (after). The triangles show the colonies poisoned at day 7, and the circles the non-poisoned colonies. High quality figures are available online.

Figure 5 shows the short-term effects of nest shading on the daily temperature difference between the fungus chamber and the soil, for both living and poisoned colonies. Before the experimental shading, the fungus chamber temperature (daily mean) for both groups exceeded that of the soil by either 5.3° C (SE = 0.6, N = 7) or 4.7° C (SE = 0.2, N = 7) (Figure 5, before shading, circles vs. triangles), values that are not statistically different (T-test, t12 = 0.89, p = 0.40). During the weeks of experimental shading, nests with living colonies still showed a fungus temperature higher than that of the soil, yet fungus chamber temperature in poisoned nests strongly decreased over time, and reached that of the soil after 14 days (Figure 5, during shading, circles vs. triangles), values that are not statistically different (T-test, t_12_ = experimental shading, nests with living colonies still showed a fungus temperature higher than that of the soil, yet fungus chamber temperature in poisoned nests strongly decreased over time, and reached that of the soil after 14 days (Figure 5, shaded, circles vs. triangles). After the wooden roofs were removed, the temperature of the nest with living colonies did not differ from that recorded before the experimental shading (Figure 5, circles, after vs. before, T-test for matched pairs, t_6_ = 1.1, p = 0.31), yet temperature in the poisoned nests was clearly lower (Figure 5, triangles, after vs. before, T-test for matched pairs, t_6_ = 1.1, p<0.001). Consequently, the temperature difference between the fungus chamber and the soil strongly differed between both treatments after removal of the roofs (Figure 5, after, circles vs. triangles, T-test, t_12_ *=* 9.95, p<0.001).

### Thermal properties of the thatch and soil

**Figure 6.**
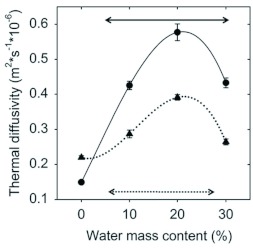
Thermal diffusivity of the thatch material and soil as a function of water mass content (%) (mean ± SE, N = 40). The black circles are the measured thermal diffusivity for the soil; the continuous line is the best-fit line regression, D_Soil_ = 0.15×10^-6^ + (0.28×10^-7^)(%) + (0.24×10^-9^)(%)^2^ - (0.29×10^-10^)(%)^3^ r^2^ = 0.91, p<0.001. The black triangles are the measured thermal diffusivity for the thatch material; the dotted line is the best-fit line regression, D_thatch_ = 0.22×10^-6^ - (0.40×10^-8^)(%) + (0.15×10^-8^)(%)^2^ - (0.45×10^-10^)(%)^3^, r^2^ = 0.73, p<0.001. The dotted and continuous straight lines show the range of soil moisture content found under field conditions for the thatch material and soil respectively. High quality figures are available online.

Thermal diffusivity of both thatch material and soil largely depended on their water mass content (Figure 6). Under extremely dry conditions, thermal diffusivities of thatch material and soil were roughly similar, yet at moderate and high water contents, the thermal diffusivity of the thatch material was clearly lower than that of the soil. Although thermal diffusivity of the thatch material increased at high water contents, probably due to the deposition of water in the air spaces that increases thermal conductivity, it was still lower than that of the surrounding soil. This occurred because at high water contents, heat capacity increased more than thermal conductivity and therefore thermal diffusivity of both, soil and thatch, decreased (Campbell 1977). Figure 6 also presents the estimated range of thermal diffusivities for field nests. They were calculated by substituting the water content of the soil and thatch samples collected in the field in the corresponding equations (Figure 6, dotted and continuous lines), and indicate that in the field, thermal diffusivity of the thatch material was lower than that of the surrounding soil.

## Discussion

### The thatch limits heat exchanges with the environment

**Figure 7.**
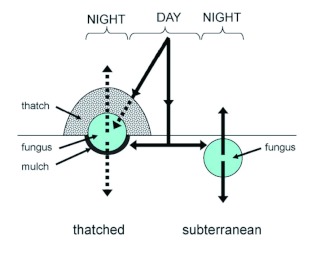
Hypothetical thermal relations between the fungus garden and the environment in a thatched nest and a subterranean nest *of Acromyrmex heyeri* during day and night. The arrows show the directions of the heat flow. The dashed and continuous lines indicate limited and non-limited heat flows, respectively. Drawings are not made at scale. High quality figures are available online.

Taken together, results indicate that a temperature surplus inside the *A. heyeri* thatched nests was largely governed by the thermal properties of the thatch material, which prevented losses of accumulated heat into the cool air at night, and avoided overheating during the day. This would not be achieved if *A. heyeri* workers built the nest mounds using the readily-available surrounding soil, since it has a greater thermal diffusivity than the grass fragments. A schematic representation of the heat flows between the environment and the *A. heyeri* nests, both thatched and subterranean, is presented in Figure 7, based on data from the present study.

During the day, the incoming solar radiation directly reached the soil and the exposed thatched nests (Figure 7, DAY, continuous line), but due to its low thermal diffusivity, the thatch material may limit the heat flow into the fungus garden (Figure 7, DAY, dashed line). In fact, the measured thermal diffusivity of the *A. heyeri* thatch, which ranged from 0.22 × 10^-6^ to 0.28 × 10^-6^ (m^2^*s^-1^) (Figure 6), was similar to that of materials considered as insulators, such as some woods (Cengel 2003), and lower than that of the mound material from *Formica polyctena* nests in the Netherlands (Brandt 1980). The observed reduction in heat flows between the nest and the environment agrees with results obtained by Frouz (2000) with *Formica polyctena* in the Czech Republic. Due to the insulation properties of the thatch material, a thatched fungus gains heat at a lower rate than a subterranean one and the environment (as shown in Figure 2, upper portion), i.e., the thatch precludes nest overheating by limiting the maximal daily temperatures that can be reached (as shown in Figure 3, lower portion).

During the night, the thatch's low thermal diffusivity prevented losses of the internally accumulated heat into the air (Figure 7, NIGHT, thatched nest). Consequently, a thatched fungus lost heat at a lower rate than a subterranean one and the environment (Figure 2, lower portion). It follows that the greater the amount of heat stored at the beginning of the cooling phase, the higher the next-day minimum (as shown in Figure 3, middle portion). Interestingly, these observations of *A. heyeri* nests indicate that fungus gardens are not directly placed on the soil in the single nest chamber, but on a 5–10 cm thick layer composed of exhausted grass fragments (mulch). This organic material is also expected to have lower thermal diffusivity than the surrounding soil (Cengel 2003), and should therefore contribute to limit the heat exchanges between the fungus garden and the underlying soil. The mulch of exhausted fungus material on which the fungus garden is placed may also prevent heat losses into the soil and so contribute to insulate the fungus garden (Figure 7, NIGHT). Since the subterranean fungus is not surrounded by insulating material, heat losses may occur throughout the cooling phase, as long as its temperature is higher than that of the surrounding soil and air (Figure 7, NIGHT, subterranean nest).

The fact that even a complete shading of thatched nests did not influence their temperature surplus clearly supports the hypothesis that it does not depend on a direct exposure to the incoming solar radiation, but is achieved by the reduced heat losses during the night (Figure 5, circles). The results with the poisoned colonies show, in addition, that the maintenance of the temperature surplus did not depend only on the properties of the nest thatch (Figure 5, triangles). Results obtained by Raigner (1948) and Korb and Linsenmair (2000) have shown for ants and termites, respectively, that the presence of the colony inside nests also contributes to the maintenance of an internal nest temperature higher than that of the environment. There are at least two possible explanations for the present results in *A. heyeri.* First, a contribution of an internal source of heat. Metabolic heat production was already shown for *Formica spp.* in the northern hemisphere, although its origin, either the colony members or the decaying nest material, is still unclear (Coenen-Stass et al. 1980; Rosengren et al. 1987; Horstmann 1990; Frouz 2000). Second, the interplay between the insulating properties of the thatch material and the high heat capacity of the fungus. The fungus may store large heat amounts during the day and lose them slowly because of the insulating properties of the thatch. Our results do not allow elucidating which of these two mechanisms may account for the high nest temperatures inside thatched *Acromyrmex* nests. Although colony poisoning acted specifically on the ants without affecting the fungus, fungus maintenance and health was probably affected by the lack of ants.

### The maintenance of temperature in thatched nests: benefits and adaptive value

Ant colonies are highly dependent of proper ranges of temperature for their growth (Porter 1988; Southerland 1988), survival (Korzukhin et al. 2001; Brian 1956) and rate of offspring production (Brian and Brian 1951; Markin et al. 1974), and workers exhibit marked thermopreferences for brood rearing. In laboratory experiments, it has been shown that *A. heyeri* preferred a temperature of 24.1° C to locate brood and fungus (Bollazzi and Roces 2002), which closely matches the temperature of 25° C at which the growth rate of the symbiotic fungus of leaf-cutting ant is maximized (Powell and Stradling 1986). Our results suggest that the temperature of a fungus garden inside a mound built hypothetically with soil, or even one located within a subterranean nest, would largely match the actual soil temperature. It follows that the average fungus garden temperature would be lower than the 24.1° C preferred by *A. heyeri* workers to cultivate the fungus garden, since soil temperature in the study area averaged values of 15.0, 17.9 and 22.7° C for winter, spring and summer, respectively, as measured over 30 years (1972 to 2002) (INIA-MGAP 2007; Van Wambeke 1981). On the contrary, a fungus garden inside a thatched nest achieves a temperature surplus of ca. 5° C regarding the surrounding soil (Figures. 4 and 5), which will positively influence rates of both fungal and brood growth.

Interestingly, the fact that the difference between fungus and soil daily maxima started decreasing beyond a maximum soil temperature of ca. 24.7° C (Figure 4) indicates that thatch building prevents a harmful overheating of the fungus garden. This thermoregulatory role of the thatch is further emphasized by the fact that maximum fungus temperature became lower than that of the soil, as soon as the soil reached ca. 30° C (Figure 4). Such a temperature matches the value at which ant brood development starts to be negatively affected, as reported for two common South American ant species inhabiting subtropical and temperate areas, *Camponotus mus* and *Solenopsis invicta* (Porter 1988; Porter and Tschinkel 1993; Roces and Núñez 1989). Furthermore, temperatures equal or higher than 30° C were found to be lethal for *in vitro* cultivars of the symbiotic fungus *Attamyces bromatificus* isolated from colonies of *Acromyrmex octospinosus, Atta cephalotes* and *Trachymyrmex urichi* (Powell and Stradling 1986). The benefits that *A. heyeri* colonies achieve by inhabiting a thatched nest is also emphasized by observations made by Bonetto (1959) in Argentina. Colonies of several *Acromyrmex* species inhabiting matched=superficial nests were observed to produce sexuals before colonies of those species inhabiting subterranean nests. Moreover, sexuals were observed earlier in nests with a thatch mostly composed of grass fragments than in those with soil in the thatch (Bonetto 1959). In the northern hemisphere, similar effects of nest location on production of sexuals have been described. Colonies of *Myrmica rubra* nesting in exposed sites were observed to produce sexuals, while those colonies nesting in shaded sites were not (Brian and Brian 1951).

The leaf-cutting ant genus *Acromyrmex* originated in southern South America during the Miocene, around 8–12 million years ago (Schultz and Brady 2008; Mayhé-Nunes and Jaffé 1998). At that time, South American climate in regions southern than 15°S started becoming dryer and seasonal, with continuously-decreasing average temperatures (Ortiz-Jaureguizar and Cladera 2006). *Acromyrmex* colonies are hypothesized to have developed adaptations to maintain their fungus gardens under proper temperatures inside their nests. In ants inhabiting soil mounds, workers track optimal rearing temperatures inside the nest and relocate the brood accordingly (Penick and Tschinkel 2008; Cole 1994). Although the material and architecture of soil mounds may allow a rapid heat gain during the day, mounds may also rapidly loose the accumulated heat at night (Cole 1994; Scherba 1962; Vogt et al. 2004; Vogt et al. 2008). The structure of thatched mounds, on the contrary, avoids losses of heat during night, as shown in the present study. Thus, this work indicates that building of a thatched nest with grass fragments instead of soil enables *A. heyeri* colonies to achieve a proper temperature range for colony growth and reproduction, emphasizing the importance of workers' building behavior for climate control in ants inhabiting temperate regions.
